# Risk factors for falls among community-dwelling older adults: A systematic review and meta-analysis

**DOI:** 10.3389/fmed.2022.1019094

**Published:** 2023-01-06

**Authors:** Ying Li, Lingyu Hou, Hanping Zhao, Rongrong Xie, Yue Yi, Xiaorong Ding

**Affiliations:** ^1^School of Nursing, Weifang University of Science and Technology, Weifang, Shandong, China; ^2^Nursing Department, Peking University Shenzhen Hospital, Shenzhen, China; ^3^Wuxi Mental Health Center, Wuxi, Jiangsu, China; ^4^Department of Neurology, Shandong Guoxin Senior Care Group Laiwu Central Hospital, Jinan, China

**Keywords:** community, older adults, risk factors, meta-analysis, systematic review

## Abstract

**Background and objective:**

The prevalence of falls among older adults living in the community is ~30% each year. The impacts of falls are not only confined to the individual but also affect families and the community. Injury from a fall also imposes a heavy financial burden on patients and their families. Currently, there are different reports on the risk factors for falls among older adults in the community. A retrospective analysis was used in this study to identify risk factors for falls in community-dwelling older adults. This research aimed to collect published studies to find risk factors for falls in community-dwelling older adults.

**Methods:**

We searched for literature from the founding of PubMed, EMBASE, the Cochrane Library, the Web of Science, the China National Knowledge Infrastructure (CNKI), the China Science and Technology Periodicals Database (VIP), and the Wanfang database until September 2022. The studies were selected using inclusion and exclusion criteria. We collected information from relevant studies to compare the impact of potential risk factors such as age, female gender, fear of falling, history of falls, unclear vision, depression, and balance disorder on falls among community-dwelling older adults.

**Results:**

A total of 31 studies were included with 70,868 community seniors. A significant risk factor for falls in the community of older adults was dementia (2.01, 95% CI: 1.41–2.86), age (1.15, 95% CI: 1.09–1.22), female gender (1.52, 95% CI: 1.27–1.81), fear of falling (2.82, 95% CI: 1.68–4.74), history of falls (3.22, 95% CI: 1.98–5.23), vision unclear (1.56, 95% CI: 1.29–1.89), depression (1.23, 95% CI: 1.10–1.37), and balance disorder (3.00, 95% CI: 2.05–4.39).

**Conclusion:**

This study provides preliminary evidence that falls among community-dwelling older adults are associated with factors such as age, female gender, fear of falling, history of falls, unclear vision, depression, and balance disorders. The results of this research may help improve clinician awareness, risk stratification, and fall prevention among community-dwelling older adults.

**Systematic review registration:**

identifier INPLASY2022120080.

## Introduction

With the advancement of society and medical and health standards, the older adults of the world (60 and older) now account for 12.3% of the total population. The average life expectancy has risen to 71 years (1). In 2018, China had 249 million people aged 60 and older, accounting for 17.9% of the total population, and approximately 167 million people aged 65 and older, accounting for 11.9% (2). The community serves as a place for older adults to live and participate in activities. According to the survey, roughly 84.5% of the older adults in the community choose a home or a combination of the community and the house for older adults.

Consequently, older adults are more inclined to fall inside the community and even at home (3). In community settings, the incidence of falls for men and women aged 65 and older ranges from 21 to 23% and from 43 to 44%, respectively (4). The community serves as a place for older adults to live and participate in activities. According to a survey, roughly 84.5% of the older adults in the community choose a home or a combination of the community and the house for older adults. Consequently, older adults are more inclined to fall inside the community and even at home (3). In Community Settings, the incidence of falls among men and women aged 65 and older was 21–23% and 43–44%, respectively (4). Falls are a leading cause of injury, related disability, and premature death among older adults (5). Injuries vary in severity, with 40–60% of falls resulting in severe lacerations, non-vertebral fractures, and traumatic head injuries (6). Prolonged lying after a fall can lead to dehydration, rhabdomyolysis, pressure sores, and pneumonia, all of which can increase hospital stays (7). Falls are also associated with successive radial, humerus, vertebral, and hip fractures (8). Approximately 95% of hip fractures are caused by falls (9). A previous study estimated that 10–20% of patients with hip fractures are admitted to nursing homes, and 20% die within 12 months (10, 11). In addition, falling can cause not only physical damage but also psychological damage (12). Falling can lead to a “fear” of falling. This “fear” can appear in 20–40% of people who fall, causing weakness and leading to a downward spiral in physical health that leads to functional decline, social isolation, and depression (12). The impacts of falls are not confined to the individual but affect families and the community. Injury from a fall also imposes a heavy financial burden on patients and their families. Fall injuries cost RMB ¥70 billion ($10 billion) in the United States and RMB ¥3.99 billion ($570 million) annually in the Netherlands, and per capita direct economic burden of China due to falls is 3,800 yuan (13–15). Falls in older adults can also result in disability, reduced quality of life, loss of independence, and hospitalization.

The risk of falls increases with the number of risk factors in each individual (16). According to the research, falls in older adults are multifactorial events involving internal (patient-related), external (environment-related), and behavioral (activity-related) aspects (17, 18). However, the sample size of each study was small, and the reported risk factors were inconsistent, so nursing professionals could not determine the risk factors for falls among older adults in the community. The meta-analysis reported pain, weakness, gait problems, dizziness, and age as risk factors for falls among older adults in the community (19–22). The reports on the relationship between poor vision and fall risk are inconsistent and controversial (23, 24). In recent years, some studies reported on the impact of risk factors such as dementia, bone and joint disease, and depression on falls of older adults in the community (25–29). However, it has yet to attract the attention of nursing professionals. To better prevent older adults from falling in the community, it is necessary to have a more comprehensive understanding of the risk factors leading to falls among older adults. This study aimed to systematically review the risk factors for falls among older adults in the community to comprehensively and systematically understand the risk factors among older adults and provide the basis for formulating relevant intervention measures.

## Methods

A meta-analysis was conducted in adherence to PRISMA (Preferred Reporting Items for Systematic Reviews and Meta-Analyses) guidelines (30).

### Data source collection

In PubMed, Web of Science, Embase, Cochrane Library, China National Knowledge Infrastructure (CNKI), Wanfang Data database, and Chinese Periodical database (VIP), a literature search was conducted to identify studies published as of September 2022 associated with risk factors for falls in older adults in the community. The search terms included “Elderly OR Aged or Older adults” and “Domicile OR Domiciles OR Community OR Communities OR Community Health Services OR Primary health care OR Characteristic, Residence OR Characteristics, Residence OR Residence Characteristic OR Residential Selection OR Residential Selections OR Selection, Residential OR Selections, Residential OR Neighborhood OR Neighborhoods OR Place of Birth OR Birth Place OR Living Arrangements OR Arrangement, Living OR Arrangements, Living OR Living Arrangement” and “Falls OR Falling OR Falls, Accidental OR Accidental fall OR fall, accidental OR Slip and Fall OR Fall and Slip” and “Risk factors OR Risk factor OR Relevant factors OR Influencing factor.” Table 1 shows the search strategy in the PubMed database. The complete search strategies for all databases are available in Supplementary material 1.

**Table 1:** Search strategy in PubMed database.

**Number**	**Search terms**	**Results**
#1	((elderly[MeSH Terms]) OR (aged[MeSH Terms])) OR (older adults[MeSH Terms])	3,423,715
#2	((((((((((((((((((((Domicile[MeSH Terms]) OR (Domiciles[MeSH Terms])) OR (Community[MeSH Terms])) OR (Communities[MeSH Terms])) OR (Community Health Services[MeSH Terms])) OR (Primary health care[MeSH Terms])) OR (Characteristic, Residence[MeSH Terms])) OR (Characteristics, Residence[MeSH Terms])) OR (Residence Characteristic[MeSH Terms])) OR (Residential Selection[MeSH Terms])) OR (Residential Selections[MeSH Terms])) OR (Selection, Residential[MeSH Terms])) OR (Selections, Residential[MeSH Terms])) OR (Neighborhood[MeSH Terms])) OR (Neighborhoods[MeSH Terms])) OR (Place of Birth[MeSH Terms])) OR (Birth Place[MeSH Terms])) OR (Living Arrangements[MeSH Terms])) OR (Arrangement, Living[MeSH Terms])) OR (Arrangements, Living[MeSH Terms])) OR (Living Arrangement[MeSH Terms])	560,689
#3	((((((falls[MeSH Terms]) OR (falling[MeSH Terms])) OR (falls, Accidenta [MeSH Terms])) OR (accidental fall[MeSH Terms])) OR (fall, accidental[MeSH Terms])) OR (Slip and Fall[MeSH Terms])) OR (Fall and Slip[MeSH Terms])	27,698
#4	(((risk factors[MeSH Terms]) OR (risk factor[MeSH Terms])) OR (relevant factors[MeSH Terms])) OR (influencing factor[MeSH Terms])	940,556
#5	(((#1) AND (#2)) AND (#3)) AND (#4)	711

### Inclusion and exclusion criteria

The inclusion criteria were as follows: (1) the study was an observational study that could be a cross-sectional, cohort, or case-control study; (2) the older adults in the community were in fact the research participants; (3) data on fall risk factors in community-dwelling older adults with 95% confidence intervals (95% CI) or odds ratios were some of the outcome types (ORs); (4) the study which met the fall definition: an unexpected event in which the participant rests on the ground, floor, or lower level; and (5) types of comparison: comparisons of fall risk factors among community-dwelling older adults.

The following types of records were excluded: (1) replicated research data; (2) incomplete data; and (3) non-original studies (conference abstracts, editorials, letters, reviews, meta-analyses, commentaries, or case reports).

### Data extraction

Two researchers independently screened the literature and extracted data according to the inclusion and exclusion criteria. In disagreements, the two parties discussed and resolved them or consulted experts. Data extraction contents included author, year, study type, number of cases and control groups, relevant risk factors, and so on.

### Statistical analysis

Review Manager 5.3 was used for statistical analysis. *I*^2^> 50% or a *P* < 0.1 was considered significant for heterogeneity (31). For homogeneous data (*I*^2^ < 50% or *P* > 0.05), the fixed-effects model was used to calculate the 95% CI and pooled ORs. The random-effects model was used in all other cases. Sensitivity analysis was carried out by eliminating one study at a time. When at least three study samples examined the same outcome measure, data were pooled and analyzed in random-effects meta-analysis models (32).

### Quality assessment

To evaluate the quality of case-control studies and cohort studies, we used Quality Assessment Scale for Non-Experimental Studies (33), based on prospective cohort studies and retrospective case-control studies, using an adapted Newcastle-Ottawa Scale (34) (the Newcastle-Ottawa Scale, NOS), including study population selection (comparability, exposure, or outcomes); cross-sectional studies were assessed using the AHRQ-recommended quality evaluation criteria (35).

## Results

### Study selection

Through the database, a total of 7,263 related literature were retrieved and a total of 2,156 repeated literature were removed. After further reading the full-text, 5,076 pieces of literature were eliminated and 31 types were finally included (25–29, 36–61), totaling 70,868 study subjects. The study and characteristics of the participants are shown in Table 2.

**Table 2:** Study and characteristics of the participants.

**Study**	**Year**	**Country**	**Type of study**	**Male/** **Female**	**N**	**Quality assessment**	**Risk factors**
Xu et al. (25)	2016	China	Cross-sectional study	656/566	1,222	8	Dementia: 1.732 (1.466, 3.835); Age: 1.459 (1.295, 1.714)
Qin et al. (26)	2006	China	Cross-sectional study	619/893	1,512	8	Dementia: 4.89 (1.31, 18.27); Fear of falling: 2.12 (1.51, 2.97)
Shi et al. (36)	2013	China	Cross-sectional study	183/289	472	7	Fear of falling: 2.23 (1.47, 3.85)
Shi et al. (27)	2016	China	Cross-sectional study	619/893	1,512	8	Female: 1.56 (1.12, 2.18); Fear of falling: 1.72 (1.41, 2.10)
Ji et al. (37)	2012	China	Cross-sectional study	495/585	1,080	8	Age: 2.395 (1.902, 3.001); Fear of falling: 1.433 (1.12, 1.833)
Xie et al. (38)	2019	China	Cross-sectional study	301/481	782	7	Age: 1.325 (1.259, 1.9); Female: 1.252 (1.131, 1.387); Vision Unclear: 3.027 (2.354, 3.636)
Shen et al. (39)	2020	China	Cross-sectional study	400/212	612	7	Age: 1.717 (1.21, 2.438); Female: 1.888 (1.328, 2.685); Osteoporosis: 3.378 (2.399, 4.755)
Wan et al. (28)	2018	China	Cross-sectional study	719/867	1,586	7	Dementia: 2.092 (1.18, 3.702) Age: 1.311 (1.114, 1.543)
Kyrdalen et al. (29)	2017	Norway	Cross-sectional study	41/67	108	8	Depression: 1.31 (1.09, 1.58); History of falls: 3.7 (1.18, 11.65)
Lastrucci et al. (40)	2018	Italy	Array research	NA	1,220	7	Age: 1.03 (1.01, 1.05)
Gamage et al. (41)	2019	Sri Lanka	Cross-sectional study	125/175	300	8	Age: 0.1 (0, 0.3); Balance disorder: 4.2 (2, 8.4)
Jindal et al. (42)	2019	India	Cross-sectional study	273/213	486	7	Depression: 1.62 (1.04, 2.51)
Stalenhoef et al. (43)	2002	Netherlands	Cross-sectional study	115/172	287	8	Age: 1 (0.5, 2.2); Female: 0.7 (0.3, 1.5); History of falls: 3.1 (1.5, 6.7)
Shi et al. (44)	2014	China	Cross-sectional study	289/173	462	7	Age: 2.2 (1.37, 3.53)
Sai et al. (45)	2001	USA	Cross-sectional study	89/48	137	7	Depression: 1.19 (1.02, 1.38); History of falls: 3.85 (1.56, 9.5)
Ooi et al. (46)	2021	Malaysia	Prospective cohort study	840/838	1,678	8	Female: 1.57 (1.04, 2.36); History of falls: 1.86 (1.19, 2.92); Depression: 1.1 (1.02, 1.2)
Carrasco et al. (47)	2018	Spain	Cross-sectional study	113/395	508	8	Female: 1.724 (1.069, 2.782); Depression: 0.763 (0.463, 1.258); Osteoporosis: 0.751 (0.414, 1.363)
Jia et al. (48)	2017	China	Cross-sectional study	1,578/1,619	3,197	8	Vision unclear: 1.43 (1.13, 1.82); Osteoporosis: 1.81 (1.04, 3.13); Balance disorder: 2.65 (1.477, 4.754)
Almada (49)	2020	Portugal	Cross-sectional study	NA	4,1098	9	Age: 1.257 (1.128, 1.402); Female: 1.313 (1.208, 1.426); Fear of falling: 3.747 (3.443, 4.078)
Almada et al. (50)	2021	Thailand	Cross-sectional study	223/239	462	7	Age: 1.07 (1.04, 1.11); Female: 3.83 (2.39, 6.13); Fear of falling: 30.09 (14.65, 61.77)
Lu et al. (51)	2016	China	Cross-sectional study	701/899	1,600	8	History of falls: 9.488 (6.544, 13.757)
Pellicer-García et al. (52)	2020	Spain	Cross-sectional study	44/169	213	7	Age: 1.092 (1.015, 1.176); Depression: 11.24 (4.169, 30.302)
Lin et al. (53)	2011	China	Cross-sectional study	704/673	1,377	8	Age: 1.03 (1, 1.06); Female: 1.94 (1.36, 2.76)
Teno et al. (54)	1990	USA	Case control study	NA	586	8	Age: 1.06 (1.02, 1.1); Female: 1.3 (0.8, 2.4); Vision Unclear: 1.7 (0.6, 4.8)
Liao et al. (55)	2012	China	Cross-sectional study	534/631	1,165	8	Age: 1.03 (1.01, 1.06); Female: 0.64 (0.46, 0.89)
Tsai et al. (56)	2020	China	Cross-sectional study	1,523/1,677	3,200	9	Age: 1.13 (0.81, 1.58);Female: 0.93 (0.72, 1.2); Vision Unclear: 1.92 (1.36, 2.72); Depression: 1.45 (1.06, 1.98)
Fong et al. (57)	2011	China	Retrospective cross-sectional study	116/483	559	7	Female: 2.5 (1.29, 4.83)
Tinetti et al. (58)	1995	USA	Cohort study	299/804	1 103	8	Female: 1.9 (1.1, 3.1)
Shuyi et al. (59)	2022	China	Prospectively study	106/145	251	7	Female: 2.71 (1.4, 5.27); Balance disorder: 2.6 (1.29, 5.24)
Leung et al. (60)	2009	China	Cross-sectional survey	619/954	1,573	8	Vision unclear: 1.29 (10.2, 1.62)
Wong et al. (61)	2013	Australia	Prospective study	258/264	520	7	Female: 1.05 (0.73, 1.49)

### Characteristics of the studies

**Figure 1:**
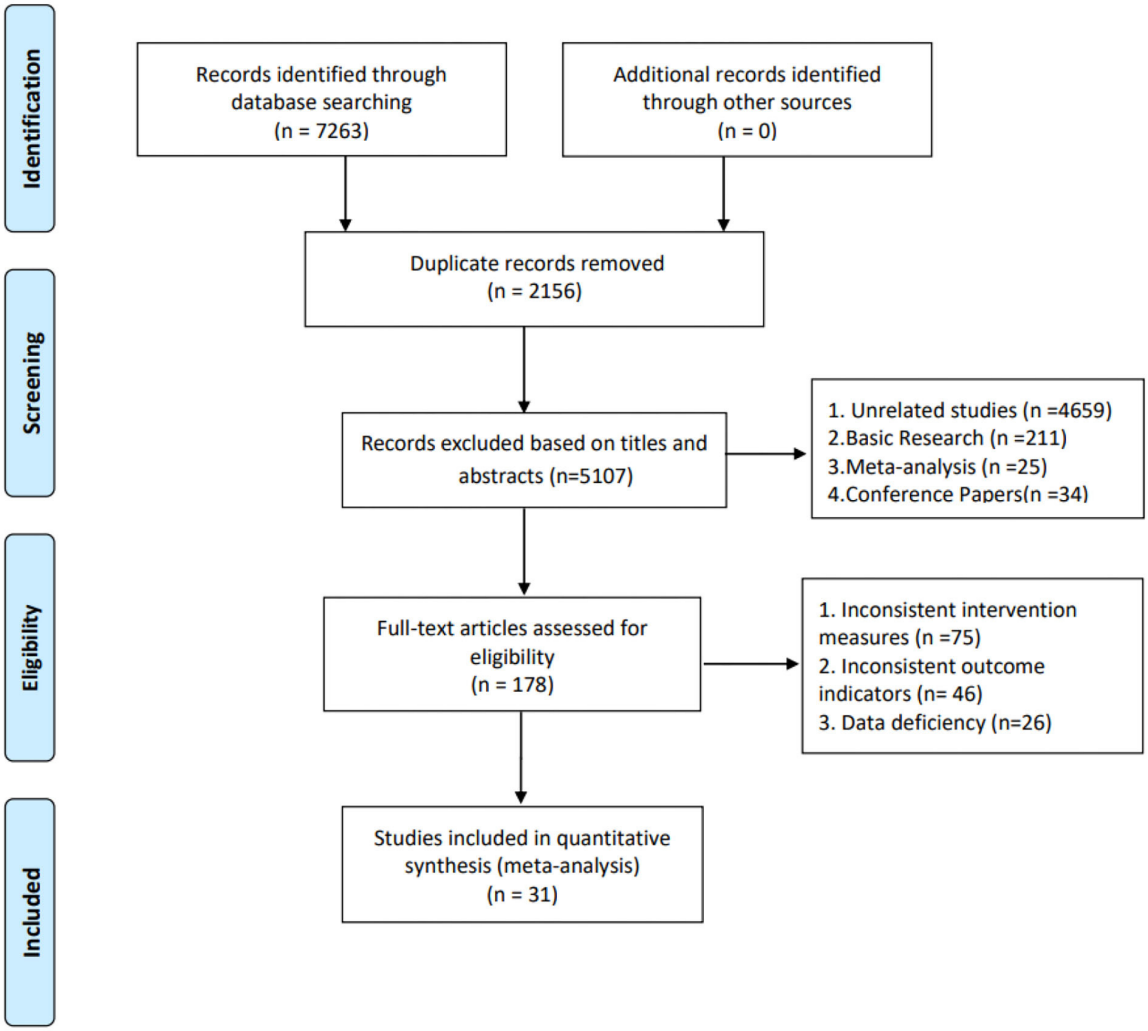
Flow diagram.

The included studies were published from 1995 to 2022: one study in Norway, one in Italy, one in Sri Lanka, one in India, one in the Netherlands, three in the USA, two in Spain, one in Australia, one in Thailand, one in Portugal, one in Malaysia, and seventeen in China. The sample sizes included in the study ranged from 251 to 41,098. The flow diagram is shown in Figure 1.

### Falls risk factors

#### Dementia

**Figure 2:**
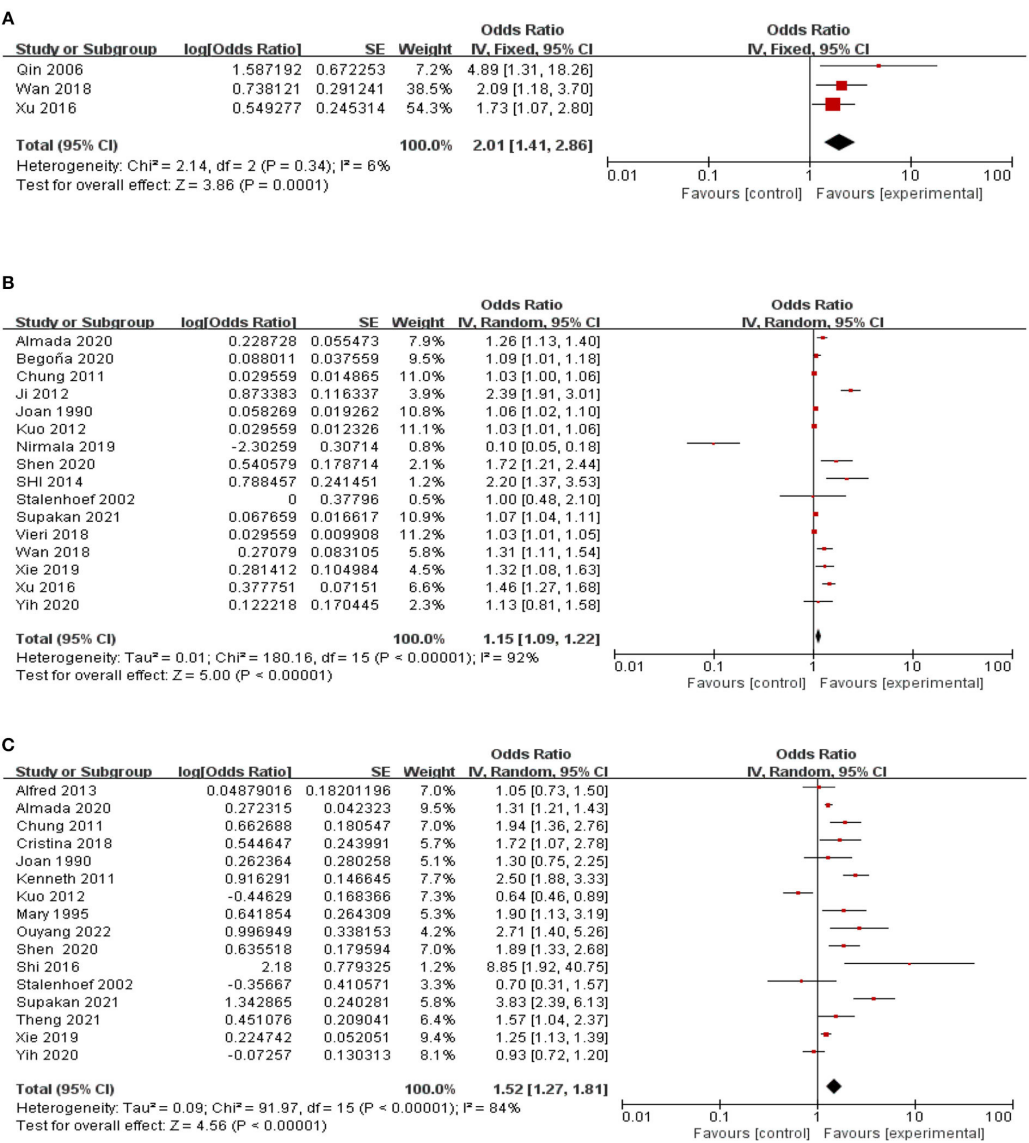
A forest plot for the association between falls among community-dwelling older adults. **(A)** Dementia. **(B)** Age. **(C)** Female.

The relationship between dementia and the risk of falls in the community was reported in three studies. Dementia significantly impacts falls (2.01, 95% CI: 1.41–2.86, Z = 3.86, *p* = 0.0001), and heterogeneity is negligible (*I*^2^= 6%) (Figure 2A).

#### Age

A link between age and the risk of falls in the community was reported in 16 studies. Age significantly affected falls (1.15, 95% CI: 1.09–1.22, *Z* = 5, *p* < 0.00001). There is a high degree of heterogeneity (*I*^2^= 92%) between studies. Therefore, these studies were grouped by region, type of study, and gender, but the results were still highly heterogeneous (Figure 2B).

### Female gender

Associations between women and the risk of falls in their communities are reported in 16 studies. Older women had a significant impact on falls (1.52, 95% CI: 1.27–1.81, Z = 4.56, *p* < 0.00001), and heterogeneity was negligible (*I*^2^= 84%). There was a high degree of heterogeneity between studies. Therefore, these studies were grouped by region and type of study, but the results were still highly heterogeneous (Figure 2C).

### Fear of falling

**Figure 3:**
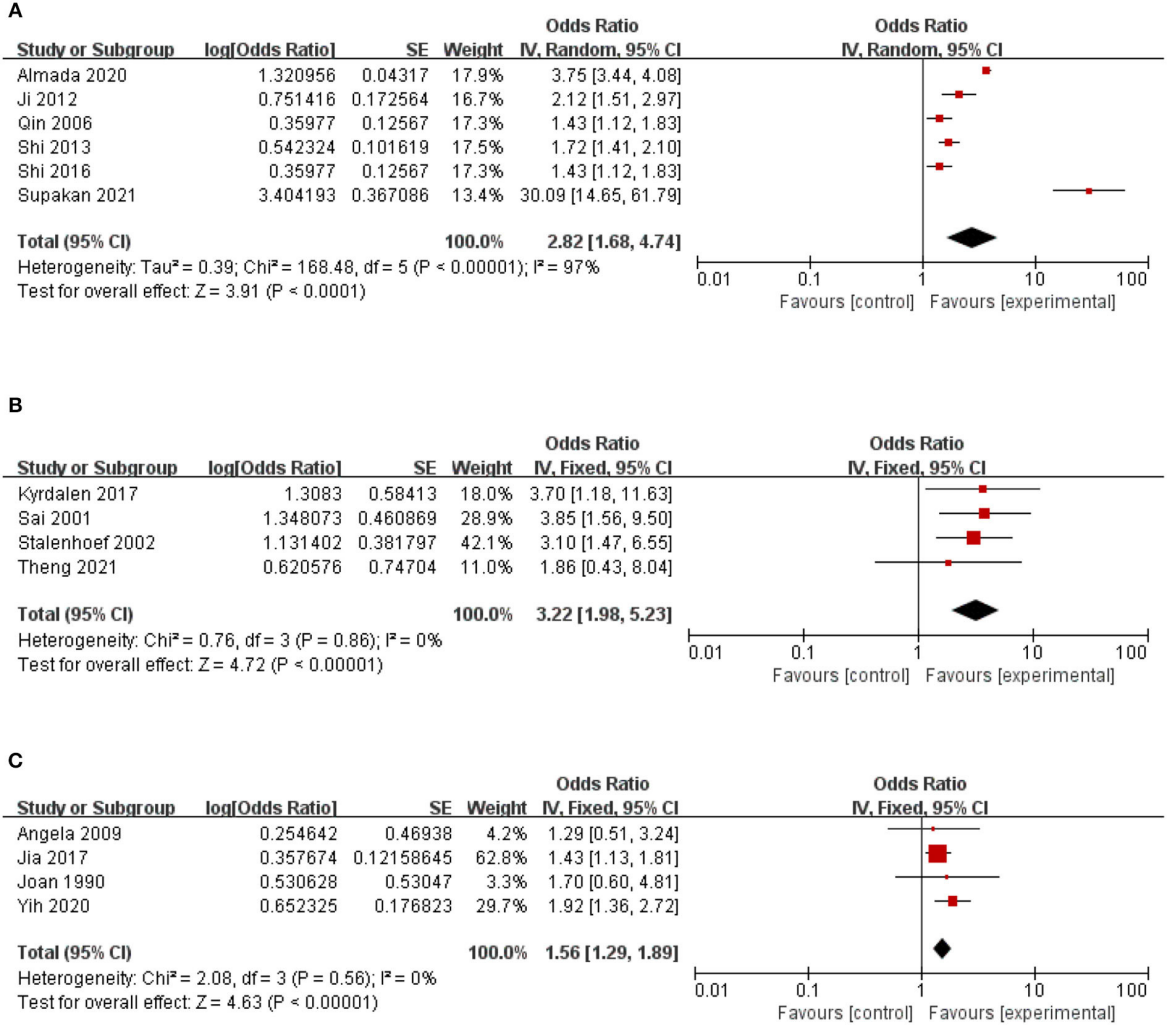
A forest plot for the association between falls among community-dwelling older adults. **(A)** Fear of falling. **(B)** History of falls. **(C)** Vision Unclear.

The relationship between fear of falling and the risk of falls in the community was reported in six studies. Fear of falling has a significant impact on falls of older adults in the community (2.82, 95% CI: 1.68–4.74, Z = 3.91, *p* < 0.0001), and there is a high degree of heterogeneity between studies (*I*^2^= 97%). Therefore, these studies are grouped by region, research type, and gender, but the results are still highly heterogeneous (Figure 3A).

### History of falls

The relationship between past falls and community fall risk was reported in five studies. The study had direct heterogeneity (*I*^2^= 68%). After sensitivity analysis, the study of Lu et al. (51) was excluded. History of falls significantly impacts the falls of older adults in the community (3.22, 95% CI: 1.98–5.23, *Z* = 4.72, *p* < 0.0001, *I*^2^= 0) (Figure 2B).

### Vision unclear

The relationship between visual impairment and fall risk in the community was reported in five studies. The study had direct heterogeneity (*I*^2^= 68%). After sensitivity analysis, the study of Xie et al. (38) was excluded. The study found that visual impairment significantly impacted falls of older adults in the community (1.56, 95% CI: 1.29–1.89, *Z* = 4.63, *p* < 0.00001, *I*^2^= 0) (Figure 2C).

### Depression

**Figure 4:**
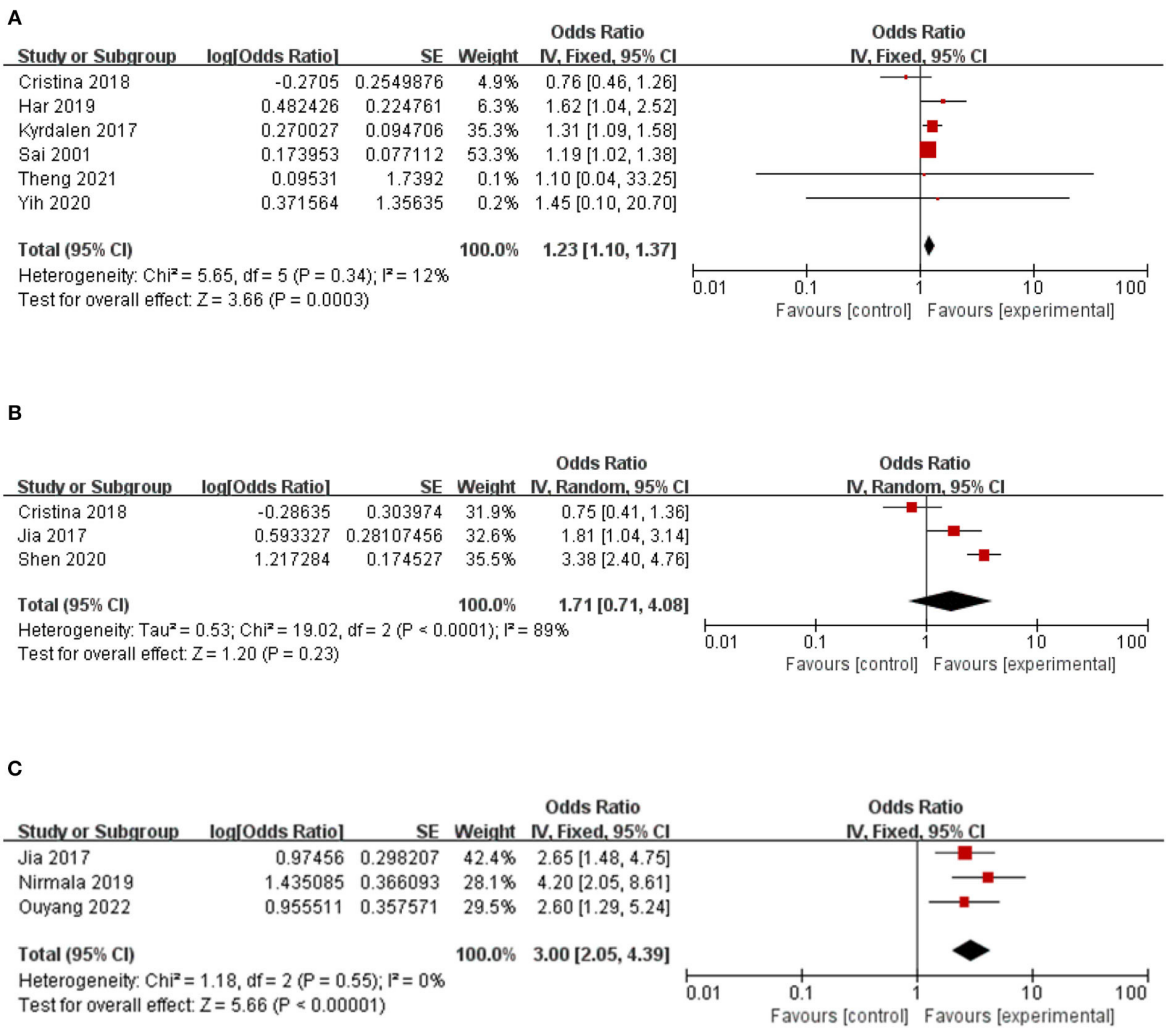
A forest plot for the association between falls among community-dwelling older adults. **(A)** Depression. **(B)** Osteoporosis. **(C)** Balance disorder.

The relationship between depression and community fall risk was reported in seven studies. The study had heterogeneity (*I*^2^=70%). After sensitivity analysis, the study of Begoña et al. (52) was excluded. The study found that visual impairment significantly impacted falls of older adults in the community (1.23, 95% CI: 1.10–1.37, *Z* = 3.66, *p* = 0.0003, *I*^2^= 12%) (Figure 4A).

### Osteoporosis

The association between osteoporosis and the risk of falls among older people in the community was reported in three studies. Osteoporosis has no significant impact on the fall risk of middle-aged and older adults in the community (1.71, 95% CI: 0.71–4.08, *Z* = 1.20, *p* = 0.23), with high heterogeneity (*I*^2^= 89%) (Figure 4B).

### Balance disorder

The relationship between balance disorders and the risk of falls in the community was reported in three studies. Balance significantly impacts falls (3.00, 95% CI: 2.05–4.39, *Z* = 5.66, *p* < 0.00001, *I*^2^= 0) (Figure 4C).

### Sensitivity analysis

In the analysis of visual impairment, history of falls, and depression, we conducted a sensitivity analysis by excluding each study one by one to explore whether a study significantly affected the results or contributed to heterogeneity. We found that the results were not affected by any research, and our meta-analysis was relatively robust. However, after excluding the studies of Xie et al. (38), Lu et al. (51), and Begoña et al. (52) the heterogeneity was significantly reduced, indicating that these two studies were the primary sources of the heterogeneity of visual impairment and depression.

### Bias assessment

**Figure 5:**
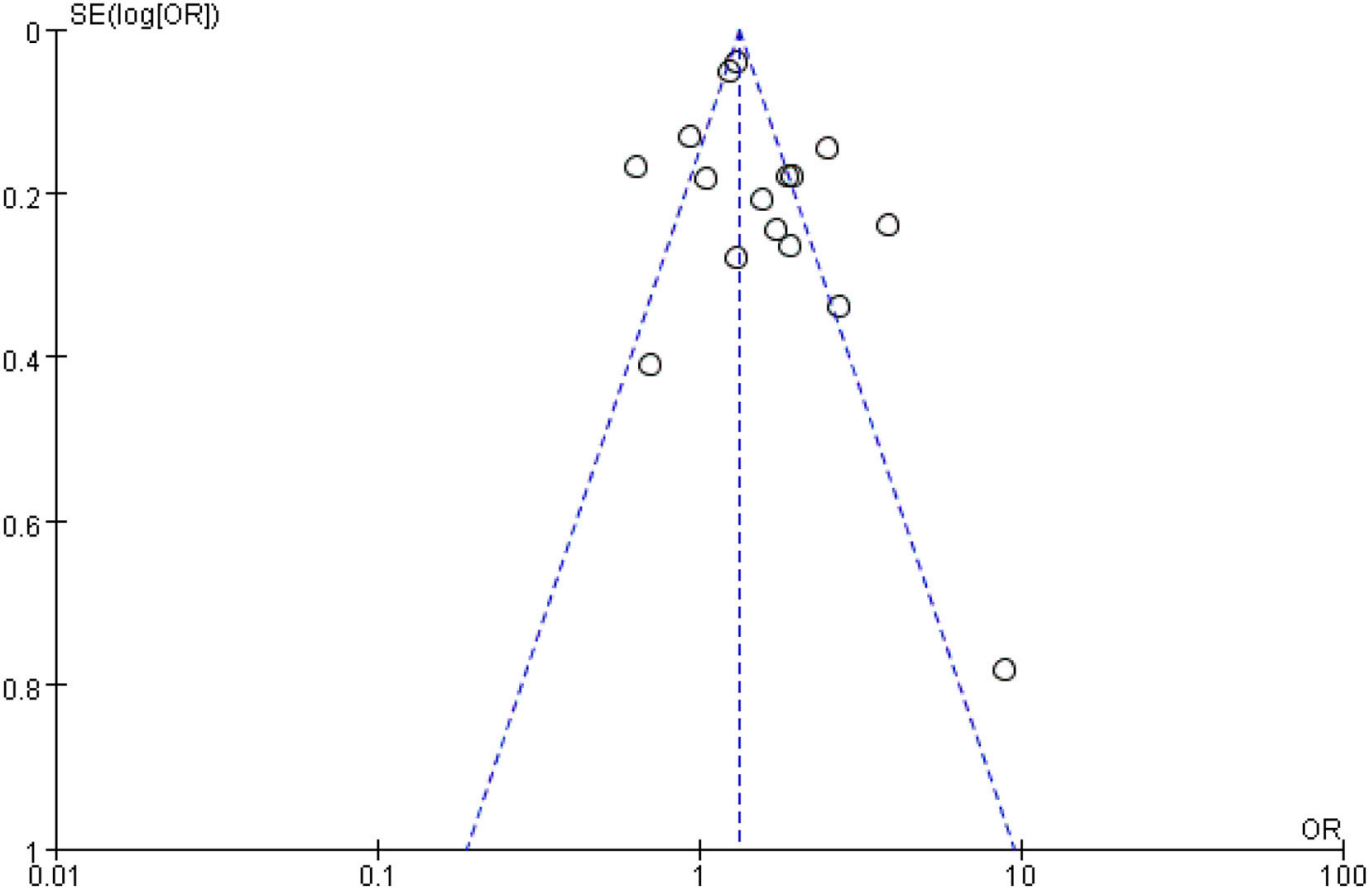
Publication bias of women.

Finally, funnel plots were constructed to qualitatively analyze the publication bias among the included studies. Fall risk factors for women and older people in the community were used as examples. The funnel diagram shows a symmetrical distribution without apparent publishing deviation (Figure 5).

## Discussion

An analysis of the included studies found that the risk factors for falls among older adults in the community are fear of falling, age, female gender, balance disorder, dementia, depression, previous falls, and unclear vision. This study found that fear of falling was closely related to falls among older adults in the community, which was consistent with the results of Sousa et al. (62). Studies showed that fear of falling and other fall-related psychological problems are common among older adults in the community and nursing homes after falls (63). The fear of falling often reduces activities in older adults, which weakens muscle strength and increases the risk of falling, increasing the fear of losing and forming a vicious circle (64). A research report pointed out that the fear of falling among older adults in the Chinese community is 41–5%, and the incidence of fear of loss among older adults in older adult care institutions is 79.4% (65, 66). According to this study, older adults should receive proper psychological intervention following a fall to reduce the psychological fear of losing and avoid falling again. This study identified age as a risk factor for falls in older people. The higher the age, the greater the risk of falling. This result is consistent with Sousa et al. (62) and Deandrea et al. (19). Because of the potential for the age-related decline, position senses, hearing, visual, and physical functions of older adults will all deteriorate. Moreover, older adults are frequently accompanied by a variety of chronic diseases. The presence of chronic diseases will impair the cognitive and balance abilities of older adults, increasing the risk of falls in older adults. Therefore, it is reasonable to believe that aging and falls among more senior adults in the community are mutually causal.

This study found balance disorders to be a risk factor for falls in community-dwelling older adults. This result was not reported in the studies of Sousa et al. (62), Deandrea et al. (19), and Chantanachai et al. (32). According to King et al., the age of 60 is a watershed in balance ability. After 60 years, the balance ability of older adults declines by 16% every 10 years, and the risk of falls also increases (67). With the increase in age, the aging of the bodies of older adults leads to weakened muscle strength and decreased joint flexibility, resulting in damage to the balance function, which will directly affect the mobility of older adults. Older adults with good balance ability have strong lower extremity muscles, high physical sensitivity, quick responses, and a low probability of falling.

In contrast, older adults with poor balance experience lower extremity weakness and reduced physical sensitivity, which increases the risk of falls (68). In addition, this study found that depression is a risk factor for falls in older adults in the community. Considering that it may be related to unstable emotions, depression weakens the attention of older adults, resulting in a decline in their ability to respond to and perceive environmental risk factors, increasing the risk of falling.

In addition, this study identified a history of falls, visual impairment, and dementia as risk factors for falls among older adults in the community. A history of falls is a risk factor among older adults in the community, and this result has not been reported in Chantanachai et al. (32) study. When comparing the older adult patients who fell in the past 6 months with the matched group who did not fall, 57% of the falls were unable to walk at the fastest speed, with short steps and small lateral swings (69). Compared with non-falling people, the variability of kinematic measurement in falling people increases (69). The changes in these actions reflect the limitation of mobility and increase the risk of falls in older adults. Older adults with a history of falls are also associated with higher symptoms of depression and anxiety (70), thus increasing the risk of falls in older adults. This study confirms that older adults with a visual impairment are 1.56 times more likely to fall than those without a visual impairment. This result is not reported in Jehu et al. (22) and Stubbs et al. (21). In the case of insufficient visual input, the ability to balance control and obstacle avoidance will be impaired due to misjudgment of distance and misunderstanding of spatial information. It has been found that impaired depth perception is one of the essential visual risk factors for older adults living in communities to fall repeatedly (71). Postural stability is a complex skill that depends on the coordination of the motor and sensory systems to perceive environmental stimuli and respond to disturbances to control body movements (72, 73). Dementia can reduce cognitive ability and gait stability, affect the ability of older adults to cope with the external environment, and increase the risk of falls in older adults.

Our findings found no association between osteoporosis and falls in older adults in the community. However, older adult patients with osteoporosis should be watched with alertness as it has been proven to be an essential risk factor for falls among older adults in the community (47). In older adult patients, calcium loss is high, and the decline of muscle content, strength, and function can significantly increase the risk of falls and fractures. Some countries, such as the United States, recommend screening for osteoporosis for all women ≥ 65 years of age (74). Therefore, we need to be more alert to osteoporosis. Similarly, the results of this meta-analysis showed that osteoporosis is not a risk factor for falls among older adults in the community, which may be because only a small sample study was included.

In conclusion, this systematic review and meta-analysis study provides strong evidence for the risk factors for falls among older adults in the community. We further confirmed that age, female gender, fear of falling, depression, visual impairment, dementia, and balance disorder increase the risk of falls for older adults in the community. In addition, the results of this study will help formulate the best practice guide for preventing falls among older adults in the community and provide a basis for establishing a prediction model.

### Limitations

There were some limitations in the present study. First, the articles included in this study were meta-integrated, but some results included only 2–3 pieces, which may have resulted in selection bias. Second, individual heterogeneity exists in the populations included in the literature of this study regarding regions, races, and socioeconomic levels. Third, because few studies involve some risk factors, meta-analysis cannot be performed, and more large-scale studies are required. Therefore, it is necessary to conduct more carefully designed studies on the potential fall risk factors in community-dwelling older adults.

## Conclusion

In conclusion, this meta-analysis identified some risk factors for community-dwelling older adults and provided a reference for preventing falls. However, more strictly designed studies are needed to substantiate our findings and identify practical measures for preventing falls.

## Data availability statement

The original contributions presented in the study are included in the article/Supplementary material, further inquiries can be directed to the corresponding author.

## Author contributions

YL and LH designed the study. RX and YY acquired, analyzed, and interpreted the data. XD, YL, YY, and HZ revised the manuscript. HZ plays the role of inspection, assistance and drafting the work or revising it critically for important intellectual content in the modification phase of the article. All authors contributed to the article and approved the submitted version.
