# Supplementary material for: Risk factors for falls among community-dwelling older adults: A systematic review and meta-analysis
Source: Front Med (Lausanne). 2023 Jan 6;9:1019094. doi: 10.3389/fmed.2022.1019094 (PMC9853191; doi:10.3389/fmed.2022.1019094)
Supplement: Supplementary file 1 [file Table_1.DOCX]

PubMed

| Number | Search terms | Results |
| --- | --- | --- |
| **#1** | ((elderly[MeSH Terms]) OR (aged[MeSH Terms])) OR (older adults[MeSH Terms]) | [3,423,715](https://pubmed.ncbi.nlm.nih.gov/?term=%28%28elderly%5BMeSH+Terms%5D%29+OR+%28aged%5BMeSH+Terms%5D%29%29+OR+%28older+adults%5BMeSH+Terms%5D%29&sort=) |
| **#2** | ((((((((((((((((((((Domicile[MeSH Terms]) OR (Domiciles[MeSH Terms])) OR (Community[MeSH Terms])) OR (Communities[MeSH Terms])) OR (Community Health Services[MeSH Terms])) OR (Primary health care[MeSH Terms])) OR (Characteristic, Residence[MeSH Terms])) OR (Characteristics, Residence[MeSH Terms])) OR (Residence Characteristic[MeSH Terms])) OR (Residential Selection[MeSH Terms])) OR (Residential Selections[MeSH Terms])) OR (Selection, Residential[MeSH Terms])) OR (Selections, Residential[MeSH Terms])) OR (Neighborhood[MeSH Terms])) OR (Neighborhoods[MeSH Terms])) OR (Place of Birth[MeSH Terms])) OR (Birth Place[MeSH Terms])) OR (Living Arrangements[MeSH Terms])) OR (Arrangement, Living[MeSH Terms])) OR (Arrangements, Living[MeSH Terms])) OR (Living Arrangement[MeSH Terms]) | 560,689 |
| **#3** | ((((((falls[MeSH Terms]) OR (falling[MeSH Terms])) OR (falls, Accidenta [MeSH Terms])) OR (accidental fall[MeSH Terms])) OR (fall, accidental[MeSH Terms])) OR (Slip and Fall[MeSH Terms])) OR (Fall and Slip[MeSH Terms]) | 27,698 |
| **#4** | (((risk factors[MeSH Terms]) OR (risk factor[MeSH Terms])) OR (relevant factors[MeSH Terms])) OR (influencing factor[MeSH Terms]) | 940,556 |
| **#5** | (((#1) AND (#2)) AND (#3)) AND (#4) | 711 |

Web of science

| Number | Search terms | Results |
| --- | --- | --- |
| **#1** | TS=(Elderly OR Aged or Older adults ) | 4,342,610 |
| **#2** | TS=( Domicile OR Domiciles OR Community OR Communities OR Community Health Services OR Primary health care OR Characteristic, Residence OR Characteristics, Residence OR Residence Characteristic OR Residential Selection OR Residential Selections OR Selection, Residential OR  Selections, Residential OR Neighborhood OR Neighborhoods OR Place of Birth OR Birth Place OR Living Arrangements OR Arrangement, Living OR Arrangements, Living OR Living Arrangement) | 1,783,332 |
| **#3** | TS=(Falls OR Falling OR Falls, Accidental OR Accidental fall OR fall, accidental OR Slip and Fall OR Fall and Slip) | 479,535 |
| **#4** | TS=(Risk factors OR Risk factor OR Relevant factors OR Influencing factor) | 2,452,043 |
| **#5** | #1 AND #2 AND #3 AND #4 | 4541 |

Cochrane Library

| Number | Search terms | Results |
| --- | --- | --- |
| **#1** | MeSH=Elderly OR Aged or Older adults | 596126 |
| **#2** | MeSH= (Domicile OR Domiciles OR Community OR Communities OR Community Health Services OR Primary health care OR Characteristic, Residence OR Characteristics, Residence OR Residence Characteristic OR Residential Selection OR Residential Selections OR Selection, Residential OR  Selections, Residential OR Neighborhood OR Neighborhoods OR Place of Birth OR  Birth Place OR Living Arrangements OR Arrangement, Living OR Arrangements, Living OR Living Arrangement) | 118795 |
| **#3** | MeSH=(Falls OR Falling OR Falls, Accidental OR Accidental fall OR fall, accidental OR Slip and Fall OR Fall and Slip) | 13475 |
| **#4** | TS=(Risk factors OR Risk factor OR Relevant factors OR Influencing factor) | 118298 |
| **#5** | #1 AND #2 AND #3 AND #4 | 1024 |

Embase

| Number | Search terms | Results |
| --- | --- | --- |
| **#1** | 'elderly'/exp OR elderly OR 'aged'/exp OR aged OR 'older adults'/exp OR 'older adults' OR (older AND ('adults'/exp OR adults)) | 5,681,314 |
| **#2** | domicile OR domiciles OR 'community'/exp OR community OR communities OR 'community health services'/exp OR 'community health services' OR (('community'/exp OR community) AND ('health'/exp OR health) AND services) OR 'primary health care'/exp OR 'primary health care' OR (primary AND ('health'/exp OR health) AND ('care'/exp OR care)) | 1,736,923 |
| **#3** | (('falls'/exp OR falls OR 'falling'/exp OR falling OR 'falls, accidental' OR (falls, AND accidental) OR 'accidental fall' OR (accidental AND ('fall'/exp OR fall)) OR 'fall, accidental' OR (('fall,'/exp OR fall,) AND accidental) OR 'slip'/exp OR slip) AND ('fall'/exp OR fall) OR 'fall'/exp OR fall) AND ('intervertebral disk hernia'/exp OR 'intervertebral disk hernia') | 240 |
| **#4** | 'risk factors'/exp OR 'risk factors' OR (('risk'/exp OR risk) AND factors) OR 'risk factor'/exp OR 'risk factor' OR (('risk'/exp OR risk) AND factor) OR 'relevant factors' OR (relevant AND factors) OR 'influencing factor'/exp OR 'influencing factor' OR (influencing AND factor) | 2,328,671 |
| **#5** | #1 AND #2 AND #3 AND #4 | 20 |

CNKI

| Number | Search terms | Results |
| --- | --- | --- |
| #1 | 老年人or 老人 | 541,748 |
| #2 | 社区 or 家中 | 1,653,463 |
| #3 | 跌倒 or 摔倒 | 37,390 |
| #4 | 危险因素or 预测因素 or 相关因素 | 1,226,804 |
| #5 | #1 AND #2 AND #3 AND #4 | 505 |

Wang Fan

| Number | Search terms | Results |
| --- | --- | --- |
| #1 | 老年人or 老人 | 270,857 |
| #2 | 社区 or 家中 | 287,718 |
| #3 | 跌倒 or 摔倒or 滑到 | 7,644 |
| #4 | 危险因素or 预测因素 or 相关因素 | 105,881 |
| #5 | #1 AND #2 AND #3 AND #4 | 52 |

VIP

| Number | Search terms | Results |
| --- | --- | --- |
| #1 | 老年人or 老人 | 371531 |
| #2 | 社区 or 家中 | 25891 |
| #3 | 跌倒 or 摔倒or 滑到 | 5,540 |
| #4 | 危险因素or 预测因素 or 相关因素 | 145690 |
| #5 | #1 AND #2 AND #3 AND #4 | 410 |
